# Evaluation of an Adaptive Game that Uses EEG Measures Validated during the Design Process as Inputs to a Biocybernetic Loop

**DOI:** 10.3389/fnhum.2016.00223

**Published:** 2016-05-18

**Authors:** Kate C. Ewing, Stephen H. Fairclough, Kiel Gilleade

**Affiliations:** School of Natural Sciences and Psychology, Liverpool John Moores UniversityLiverpool, UK

**Keywords:** psychophysiology, EEG, gaming, physiological computing, adaptive interface, effort, engagement

## Abstract

Biocybernetic adaptation is a form of physiological computing whereby real-time data streaming from the brain and body is used by a negative control loop to adapt the user interface. This article describes the development of an adaptive game system that is designed to maximize player engagement by utilizing changes in real-time electroencephalography (EEG) to adjust the level of game demand. The research consists of four main stages: (1) the development of a conceptual framework upon which to model the interaction between person and system; (2) the validation of the psychophysiological inference underpinning the loop; (3) the construction of a working prototype; and (4) an evaluation of the adaptive game. Two studies are reported. The first demonstrates the sensitivity of EEG power in the (frontal) theta and (parietal) alpha bands to changing levels of game demand. These variables were then reformulated within the working biocybernetic control loop designed to maximize player engagement. The second study evaluated the performance of an adaptive game of Tetris with respect to system behavior and user experience. Important issues for the design and evaluation of closed-loop interfaces are discussed.

## Introduction

Biocybernetic control describes how the implicit measurement of physiological signals from the brain or body can be transformed into a control input for real-time software adaptation. This category of physiological computing system (Fairclough, 2009) has also been described as a passive brain-computer interface (Zander and Kothe, 2011) because the user simply responds to events at the interface without any requirement for volitional control. The purpose of biocybernetic adaptation is to create a seamless and tacit form of human-computer interaction where software adaptation is timely and intuitive from the perspective of the user.

The biocybernetic model has been applied to a range of domains, such as: adaptive automation (Bailey et al., 2006), detection of negative emotions (Kapoor et al., 2007), adaptive robotics (Liu et al., 2007) and support for social behavior (Chanel and Mühl, 2015). An early example of a working biocybernetic control loop was developed by NASA in the 1990s where the real-time analysis of electroencephalography (EEG) signals was converted into an input variable for the control of the level of system automation during simulated aviation tasks (Pope et al., 1995; Freeman et al., 1999; Prinzel et al., 2000; Scerbo et al., 2003). This control loop was designed to sustain operator engagement within an optimal zone that avoided complacency and inattention by selectively disabling system automation in order to oblige the operator to engage with a manual interface. This example of biocybernetic control set a blueprint for a data processing protocol wherein electrocortical activity interacts with a computerized system within a negative feedback loop. This model of closed-loop control detects deviations from an optimal state of brain activity and uses these variations to cue changes at the human-computer interface in order to “pull” the psychological state of the user in a desired direction.

The design of a biocybernetic closed-loop incorporates a number of distinct processing stages: (1) data collection from sensors, (2) filtering of raw data coupled with artifact correction techniques, (3) data analysis for the extraction of meaningful metrics that permit a valid inference of the user state, (4) conversion of the metrics in order to instigate adaptation at the user interface, i.e., defining criteria/triggers for adaptation or by categorizing data using machine learning algorithms (Baldwin and Penaranda, 2012; Novak et al., 2012); and (5) adaptation of the user interface in a manner designed to promote a desirable user state.

All biocybernetic closed-loop systems are rooted in a psycho-physiological inference; for example: inferring increased arousal from increases in skin conductance level, inferring negative affect from activation of the corrugator supercilli. The validity of this inference is fundamental to the integrity of a working loop, but the process of establishing validity is complex (Cacioppo and Tassinary, 1990). The loop is designed to utilize software adaptation in order to influence a key psychological concept or dimension in the user, e.g., engagement, mental workload, attention. If the fundamental link between input measures, the psychological concept targeted by those measures and the adaptive logic of the loop is weak or tenuous, then the effectiveness of the closed-loop system will be compromised (Fairclough, 2007, 2009). Because the loop works in real-time, it is important that measures are: (a) sufficiently sensitive to changes in the relevant psychological dimension; and (b) specific to that dimension, i.e., not confounded with other psychological variables. Consequently it is important to construct biocybernetic loops on the basis of measures that have either been scientifically validated according to research literature or tested and validated in the context of the target task or application.

This article describes the development of an adaptive computer game where the software responds in real-time in order to enhance the experience of the player by making the game appropriately challenging. Optimizing task difficulty is one of several methods of adapting gaming experiences using biocybernetics, others include enhancing emotional engagement and reducing player frustration (Gilleade et al., 2005). This closed-loop approach employs the same logic that underpins the integration of biofeedback mechanics into gaming applications (Nacke et al., 2011) and the design of adaptive games dedicated to the creation of a specific emotion (Dekker and Champion, 2007). One goal for an adaptive game is to deliver a level of difficulty tailored to the skills of the player via closed-loop control such that the game is personalized to the skills and abilities of each player. This article will describe the development of an adaptive game of Tetris designed to sustain player engagement (see also Chanel et al., 2011) and also an experimental study intended to validate the psychophysiological inference underpinning the system that was conducted prior to the creation of the working prototype.

**Figure 1:**
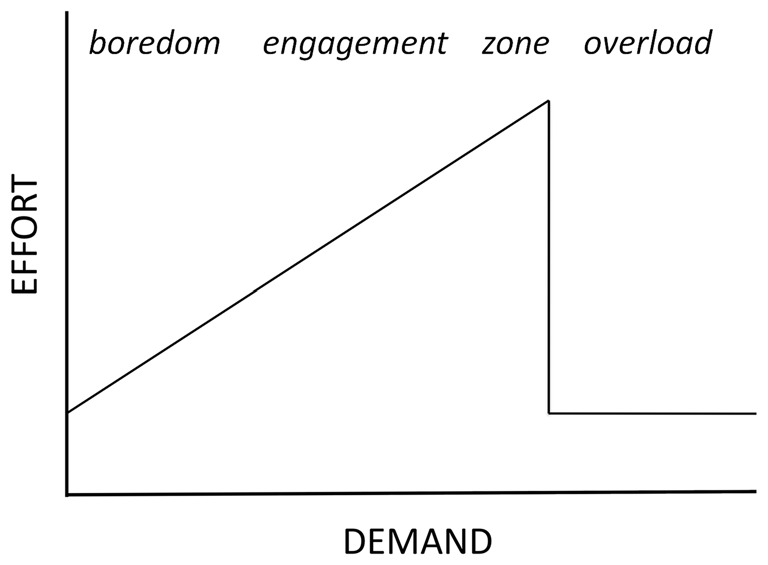
**Motivational Intensity Model (MIM) adapted by the addition of four categories of user state**.

Game construction began with the formulation of a conceptual framework upon which to model the responses of the adaptive game. Our framework was based upon the Motivational Intensity Model (MIM: Wright, 2008) which describes the relationship between effort investment and task demand; a model that has been corroborated via a number of experimental studies (e.g., Wright and Kirby, 2001; Richter et al., 2008; Richter and Gendolla, 2009). One prediction of this model is that effort rises proportionally with increases in task difficulty until demand is so great that the human deems task success unlikely and withdraws effort, the result of which is a shark-fin shaped effort curve (Figure 1). The MIM was adapted to provide a conceptual framework for defining a desirable state of player engagement that could serve as the target for the biocybernetic loop. The adaptation took account of research upon the gaming experience to define an ideal “zone” state for the player. For instance, Csikszentmihalyi (1990) described the ideal or optimal level of engagement as “flow”; a state where engagement with a task is full to the point that time seems to slip away. According to Nacke and Lindley (2008) flow is characterized by an absence of undesirable mental states (i.e., boredom) and entails a positive emotional experience. Similar states, such as being in the zone or total immersion have been described by Chen (2004) and Ryan et al. (2006) respectively. The observation has also been made that situations of high effort promote skill development and an opportunity to demonstrate mastery or competence that leads to a positive gaming experience (Ryan et al., 2006). Thus, the MIM was adapted to represent four broad categories of player state; boredom, engagement, zone and overload (Figure 1). The conceptual distinction between these four categories was used to define adaptive goals for the biocybernetic loop, namely:

- To avoid boredom by increasing game demand whenever boredom was detected
- To reduce demand when overload was detected
- To make no adjustment when the player occupied the target states of engagement and zone

In order for the control loop to work within this framework (Figure 1) the model must be operationalized using psychophysiological measures. The MIM has been extensively corroborated by cardiovascular indices of mental effort (e.g., Wright and Dill, 1993; Wright and Kirby, 2001; Richter et al., 2008; Richter and Gendolla, 2009), however cardiovascular measures have a number of limitations as inputs to a biocybernetic loop including an inability to diagnose and monitor individual psychological dimensions of effort, e.g., reactivity in blood pressure is simultaneously sensitive to motivation, cognitive effort and physical effort (Cacioppo et al., 2000). By contrast EEG provides a wide choice of metrics that permit a multidimensional monitoring of engagement, including spontaneous oscillations, evoked and event-related potentials (EPs and ERPs), different frequency bands, scalp locations and power values. Multivariate combinations of EEG measures have demonstrated impressive levels of accuracy at discriminating user workload (e.g., Gevins et al., 1998; Prinzel et al., 2003; Scerbo et al., 2003; Chanel et al., 2008; Christensen et al., 2012). Of particular interest are EEG oscillations in the alpha (7.5–13 Hz) and theta (4–7 Hz) bands, which are reliable measures of cortical activation and mental effort (e.g., Gevins et al., 1998; Klimesch, 1999; Wilson, 2002, 2003). In an earlier study (Fairclough et al., 2013), measures of power in the alpha and theta bands were sensitive to manipulations of cognitive demand and motivational incentives using the *N*-back working memory task, however, the capacity of these metrics to index demand and motivation in the context of a computer game remained unknown.

## Study One: Validation of Input Measures

### Introduction

An experimental study was conducted to evaluate the sensitivity and reliability of the EEG alpha and theta bands to variations in game demand and motivation during the play upon the popular game Tetris. The study aimed to establish: (a) the most suitable EEG measures to use as inputs to a real-time biocybernetic loop; and (b) an appropriate framework for the operationalization of the MIM with respect to measures of spontaneous EEG. The study employed a within subjects design and involved game based manipulations of motivation and demand: three levels of game demand were tested (low, high, excessive) along with two incentive conditions whereby a game-based incentive was present in one condition and absent in the other. It was expected that changes in oscillatory EEG activity in the alpha and theta bands would capture: (1) situations of low effort (i.e., due to boredom or overload); (2) instances of effort increasing in line with demand (when players were engaged with the game) and most significantly; and (3) when players were in the “zone” (when maximal effort was apparent; Figure 1). It was also anticipated that the addition of an incentive would increase effort investment provided that game success was likely (Wright, 2008).

### Method

#### Participants

Twenty participants (11 females) took part in the experiment. Participants were aged between 19 and 36 years, and had a mean age of 23.2 years (*SD* = 4.02). All participants were volunteers who gave their written informed consent prior to data collection in accordance with the Declaration of Helsinki.

#### Game Demand

**Figure 2:**
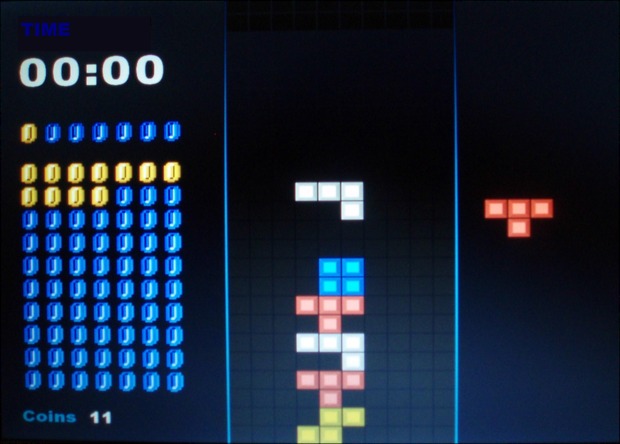
**Game-board during incentive + feedback condition: coins are displayed pictorially in a 7 × 10 matrix on the left of the screen and turn from dark blue to gold to indicate coin achievement.** A separate row of coins above indicates the number of coins awaiting award at the next 10 s time-point (one coin in this example). The coin score (bottom) and remaining game time are presented in numerals on the left of the screen.

Cognitive demand was manipulated using an adapted version of the Tetris game. The game requires participants to rotate and move falling pieces in order to build rows of blocks at the bottom of a game board. Falling pieces were one of seven possible colored shapes; each comprised of four squares arranged in different configurations. Pieces were selected to fall in random order. In order to allow gameplay for a fixed duration of 180 s the conventional Tetris game-board was adapted to prevent game-death (when pieces stack to the top of the board to signal game-over). The adaptation consisted of shifting the game-board upwards so that the highest stacked piece was maintained at the center of the game board, and was unable to rise above this level (Figure 2).

The speed and quantity of the falling pieces were systematically manipulated to create three levels of game demand (low, high or excessive). In the low demand condition, an average of 22.1 pieces fell with a drop speed of 2.5 board squares s^−1^ An average of 66.2 pieces fell with a drop speed of 6.7 board squares s^−1^ in the high demand condition. In the excessive demand condition, an average of 217 pieces fell at a drop speed of 20 board squares s^−1^. These parameters were determined on the basis of a small pilot study (*N* = 7).

#### Incentives

Games were presented in one of two incentive conditions (incentive + performance feedback vs. no incentive + no performance feedback). Each participant completed both incentive conditions (i.e., within-subjects). In the incentive + feedback condition, game coins could be earned for completing rows of Tetris pieces. Coins were accrued in proportion to the number of rows cleared relative to the maximum possible row clearance, such that a maximum of 70 coins could be earned (representing 100% possible clearance). Between zero and seven coins were accumulated every 10 s depending on the proportion of maximum cleared rows achieved at the time of accrual, i.e., at the end of each game, best performance = 70 coins and worst performance = 0 coins (Figure 2). Sounds were presented with each award of coins: “kerching” with an award (if current total was less than 35 coins) or “coin jackpot” (if total was over 35 coins). In the no-incentive (+ no feedback) condition, the display related to the coin incentive was absent and no sound effects related to the award of coins were played. For both incentive conditions sound effects occurred when rotating the pieces (small “pop”) and shifting the pieces left or right (small “snap”).

#### Experimental Design

The experiment consisted of six 180 s games (2 incentive blocks × 3 levels of demand per incentive block). Incentive blocks were delivered in a counterbalanced order and each level of demand presented in random order within each incentive block. *Post hoc T*-tests questionnaires were completed after each game. Throughout each game EEG was measured along with task performance; the total duration of the experimental session was approximately 40 min. Participants practiced by playing each of the six game versions once prior to the experiment and the fitting of EEG equipment. The procedure for the experiment and data collection protocol was approved by the Liverpool John Moores University (LJMU) University Research Ethics Committee and the experiment was conducted in accordance with the recommendations of the LJMU University Research Ethics Committee.

#### Subjective Questionnaires

Subjective workload was assessed using the NASA Task Load Index (TLX; Hart and Staveland, 1988) which consists of six scales (subjective effort, mental demand, temporal demand, physical demand, perception of performance and frustration). Subjective levels of motivation were assessed using the Dundee State Stress Questionnaire (DSSQ) v1.2 motivation scale, which includes eight items relating to motivation, task enjoyment, desire for success, task value, mental effort, agreeableness on completion, concern over poor performance and eagerness to do well (Matthews et al., 1999). Participants completed one version of each questionnaire immediately after each of the six experimental conditions.

#### EEG Recording and Analysis

EEG was recorded monopolarly from 64 Ag–AgCl pin-type active electrodes mounted in a BioSemi stretch-lycra head cap. Electrodes were positioned using the international 10–20 system and recorded activity from the following sites: frontal pole (FPz, FP1 and FP2), anterior-frontal (AFz, AF3, AF4, AF7 and AF8), frontal (Fz, F1, F2, F3, F4, F5, F6, F7 and F8), fronto-central (FCz, FC1, FC2, FC3, FC4, FC5 and FC6), central (Cz, C1, C2, C3, C4, C5 and C6), temporal (FT7, FT8, T7, T8, TP7 and TP8), parieto-central (CPz, CP1, CP2, CP3, CP4, CP5 and CP6), parietal (Pz, P1, P2, P3, P4, P5, P6, P7, P8, P9 and P10), occipito-parietal (POz, PO3, PO4, PO7 and PO8) and occipital/inion (Oz, O1, O2 and Iz). Two reference electrodes, the “common mode sense” (CMS) and “driven right leg” (DRL) were used; these function via a feedback loop to drive the participant’s voltage (acquired via CMS) as close as possible to zero. AC differential amplifiers performed continuous digitization at 16,384 Hz which was then down-sampled online to 256 Hz. No filters were applied online to allow visual inspection of noise. Offline filtering was performed using a notch filter of 50 Hz and high and low pass filters of 0.05 and 40 Hz respectively. The data were visually inspected for artifacts from external electromagnetic sources. Automatic correction of blink artifacts and horizontal and vertical saccades was performed using detection through predefined topographies. Muscle activity over 100 μV was also excluded. Fast fourier transforms (FFTs) were computed over 50% overlapped windows of 2 s (512 points). The total power in μV^2^ was obtained for lower alpha frequency band (7.5–10 Hz), upper alpha frequency band (10.5–13 Hz) and theta frequency band (4–7 Hz; Klimesch, 1999). For the analysis of spectral power in the alpha bands data from the electrodes most spatially representative of the regions of interest were used i.e., frontal (F3, F4); temporal (T7, T8); central (C3, C4); parietal (P3, P4); occipital (O1, O2). This selection permitted analysis of distributed signals whilst minimizing type one error. The theta band used in this study consisted of a 1 Hz window taken around the frequency of peak modulation within the 4–7 Hz theta range for each participant. This was in order to individualize measurements and maximize their validity. As the majority of participants tend not to produce a clear peak frequency within the theta band, and because there tends to be a large inter-individual variability in the magnitude of the theta response to demand, individualization of the measure was deemed necessary (Gevins et al., 1998). The method involved (for each participant) plotting the spectral power values that lay within the 4–7 Hz theta band for each demand condition on a graph where frequency was represented on the *x*-axis and spectral power on the *y*-axis. The graph for each participant was then visually inspected to discern the theta frequency possessing the greatest demand related modulation of power. Many participants did not display a unique frequency with the greatest power modulation, but instead a small window of similar frequencies that displayed greater modulation than the other theta frequencies; for this reason a 1 Hz window was selected for each participant. Power spectra values for both alpha and theta bands were log transformed (using the natural log) to normalize distribution. A single 180 s continuously recorded data segment was analyzed for each experimental condition.

#### Statistical Analysis

*A priori* hypotheses concerning effects for demand were tested using repeated measures analyses of variance (ANOVA). Multivariate analyses are reported using the Pillai’s Trace statistic and where multivariate tests failed to reach significance, due to a small sample size (*N* = 20) significant univariate analyses are reported. Greenhouse-Geisser corrections were applied for violations of sphericity as indicated by Mauchly’s test. Alpha levels for *a priori* tests were set at 0.05. Significant omnibus effects have been followed up with *post hoc* tests where the alpha levels were corrected to minimize Type one errors using the Bonferroni adjustment.

### Results

#### Performance

A 2 × 3 (incentive × demand) repeated measures ANOVA was performed on game performance scores (i.e., the percentage of successful line completions), which revealed an omnibus effect for demand (*F*_(2,18)_ = 504.8, *p* < 0.01, *η*^2^ = 0.98). There were no main or interaction effects for the incentive. *Post hoc* tests revealed that performance was significantly reduced at excessive compared to high demand (*p* < 0.01) and low demand (*p* < 0.01). Performance scores were also significantly lower at high compared to low demand (*p* < 0.01), descriptive statistics are presented in Table 1.

**Table 1:** **Mean scores and standard deviation (in brackets) for Tetris performance (the percentage of rows completed; *N* = 20)**.

Demand	Low		High		Excessive	
Incentive	Inc.	No inc.	Inc.	No inc.	Inc.	No inc.
Performance	**70.67**	**63.44**	**58.59**	**49.63**	**2.98**	**2.41**
	(12.67)	(13.43)	(26.9)	(28.55)	(3.26)	(2.35)

#### Subjective Self-Report Data

A 2 × 3 (incentive × demand) MANOVA on scores for the six scales of the NASA TLX revealed significant main effects for demand (*F*_(12,220)_ = 22.64, *p* < 0.01, *η*^2^ = 0.55) and incentive (*F*_(6,109)_ = 2.85, *p* < 0.05, *η*^2^ = 0.14). Ratings of mental, physical and temporal demand increased significantly with each increment in demand (all *p* < 0.05). Effort ratings increased from low to high demand (*p* < 0.01) and showed a marginally significant increase at excessive vs. high demand (*p* = 0.05). Perceptions of performance quality were reduced at excessive vs. high and low demand (both *p* < 0.01) while frustration was elevated at excessive vs. high and low demand (both *p* < 0.01). Ratings of mental demand, physical demand and effort all increased with incentive (*p* < 0.05). However there was no effect for incentive upon the ratings of temporal demand, frustration and perception of performance quality; descriptive statistics are provided in Table 2.

**Table 2:** **Mean and standard deviation (brackets) scores for the six NASA TLX Scales (mental demand, physical demand, temporal demand, frustration, effort and perception of performance) and the DSSQ motivation scale**.

Demand	Low		High		Excessive	
Incentive	Inc.	No inc.	Inc.	No inc.	Inc.	No inc.
Mental demand	3.77 (2.29)	3.00 (2.17)	6.50 (1.87)	5.32 (2.03)	7.73 (1.95)	7.00 (2.77)
Physical demand	3.00 (2.25)	2.09 (1.51)	5.50 (2.8)	4.05 (2.58)	6.73 (2.79)	7.00 (2.77)
Temporal demand	2.00 (0.87)	1.95 (1.48)	6.09 (1.45)	6.00 (1.8)	9.27 (1.13)	8.72 (1.92)
Frustration	4.18 (2.58)	3.95 (2.42)	4.18 (2.33)	4.36 (2.53)	8.18 (2.02)	7.64 (2.4)
Effort	4.41 (2.4)	2.95 (1.93)	7.23 (1.78)	5.68 (2.24)	8.41 (1.53)	6.77 (2.61)
Perception of performance	7.27 (2.54)	7.55 (2.05)	7.14 (2.41)	5.73 (2.64)	1.50 (0.99)	2.23 (1.78)
Motivation	6.14 (0.91)	4.86 (1.88)	7.60 (1.04)	6.20 (1.38)	5.73 (1.68)	5.40 (1.31)

*Inc., incentive; No inc., no incentive; N = 20*.

Scores on items from the DSSQ Motivation subscale had a high internal consistency (Cronbach’s alpha = 0.88) so were collapsed into one index of subjective motivation. A demand (3) × incentive (2) repeated measures ANOVA revealed significant main effects for demand (*F*_(2,18)_ = 29.42, *p* < 0.01, *η*^2^ = 0.77) and incentive (*F*_(1,19)_ = 15.16, *p* < 0.05, *η*^2^ = 0.44). *Post hoc T*-tests indicated enhanced motivation at high demand (high vs. low: *p* < 0.01; high vs. excessive: *p* = 0.01). Motivation was also elevated when the incentive was present for all demand conditions (*p* = 0.01; Table 2).

#### EEG Theta Power

A 2 × 3 repeated measures MANOVA was conducted on theta power data from five frontal (F, FC) and AF sites (AFz, Fz, FCz, F1, F2). This analysis produced a main effect for demand (*F*_(2,18)_ = 21.89, *p* < 0.01, *η*^2^ = 0.71) and site (*F*_(4,16)_ = 38.73, *p* < 0.01, *η*^2^ = 0.91). A quadratic trend for demand was significant (*F*_(1,19)_ = 19.71, *p* < 0.01, *η*^2^ = 0.51) indicating maximum power at high demand. There was no effect of incentive on frontal theta power.

To locate the effects for demand paired sample *T*-tests were conducted on data that had been collapsed across the levels of site and incentive. Theta power was significantly elevated at high vs. low and excessive demand (*p* < 0.01). There was also a marginally significant increase of theta power during excessive compared to low demand (*p* = 0.05).

#### EEG Alpha Power (7.5–13 Hz)

To discern effects of the manipulations upon spectral power in the alpha band, repeated measures (2 × 3 × 5 × 2) ANOVAs with factors of incentive (incentive, no incentive) × demand (low, high, excessive) × site (frontal (F3, F4), parietal (P3, P4), occipital (O1, O2), central (C3, C4), temporal (T7, T8)) × hemisphere (left, right) were performed separately on lower and upper alpha band power.

The omnibus analyses for lower alpha band power (7.5–10 Hz) produced main effects for site (*F*_(4,16)_ = 41.05, *p* < 0.01, *η*^2^ = 0.91) and hemisphere (*F*_(1,19)_ = 4.92, *p* < 0.04, *η*^2^ = 0.21). Trend analysis showed a linear trend for hemisphere with reduced lower band power in right hemisphere (statistic as for effect). Interactions were also present in the analysis of lower alpha power for incentive × hemisphere (*F*_(1,19)_ = 5.73, *p* < 0.03, *η*^2^ = 0.23) and demand × site (*F*_(4,82)_ = 4.01, *p* < 0.01, *η*^2^ = 0.17). *Post hoc* tests indicated the incentive × hemisphere interaction was related to greater reduction of alpha power in right hemisphere during the incentive condition (*p* = 0.02). The demand × site interaction was linked to a reduction of lower alpha power at occipital sites during high compared to excessive demand (*p* = 0.03); lower alpha was also suppressed at high compared to low demand at temporal sites (*p* < 0.01). Summary statistics for the *post hoc* tests are presented in Table 3.

**Table 3:** **Differences in power between levels of Tetris demand by region for lower alpha band (*N* = 20)**.

	Lower alpha band power			
Site		*t*	*p*	*η*^2^
Occipital	excess > high	2.29	0.03	0.22
Temporal	high > low	2.92	0.01	0.31

The omnibus ANOVA for upper alpha band (10.5–13 Hz) produced main effects for incentive (*F*_(1,19)_ = 6.41, *p* < 0.03, *η*^2^ = 0.25), demand (*F*_(2,18)_ = 6.62, *p* < 0.01, *η*^2^ = 0.42) and site (*F*_(4,16)_ = 25.22, *p* < 0.01, *η*^2^ = 0.86). There were significant linear trends indicating that upper alpha power decreased as demand increased (*F*_(1,19)_ = 13.63, *p* < 0.01, *η*^2^ = 0.42) and when the incentive was offered (statistic as for effect). Interactions were also present for incentive × hemisphere (*F*_(1,19)_ = 6.81, *p* < 0.02, *η*^2^ = 026) and demand × site (*F*_(4,81)_ = 8.69, *p* < 0.01, *η*^2^ = 0.31).

*Post hoc T*-tests revealed a reduction of upper alpha power when game coins were present (*p* = 0.02). Upper alpha was also suppressed at excessive compared to high and low demand (*p* < 0.01) and at high compared to low demand (*p* = 0.02) indicating a concomitant drop in upper alpha power as game demand increased.

Analysis of the demand × site interaction revealed a stepwise reduction of upper alpha power as demand increased at parietal, frontal and central sites. However, this demand effect was not apparent at occipital and temporal sites. *Post hoc* tests indicated that the hemisphere × incentive interaction was related primarily to a reduction in power during the incentive condition compared to the no-incentive condition in the right hemisphere (*p* < 0.01). The *t*-values and effect sizes for these *post hoc* tests are displayed in Table 4.

**Table 4:** **Differences in power between levels of Tetris demand by region for upper alpha band (*N* = 20)**.

	Upper alpha band power			
Site		*t*	*p*	*η*^2^
Central	low > excess	5.14	<0.01	0.58
	low > high	4.72	<0.01	0.54
	high > excess	3.95	0.01	0.45
Parietal	low > excess	4.24	<0.01	0.49
	low > high	3.55	0.02	0.40
	high > excess	3.18	<0.01	0.35
Frontal	low > excess	4.18	<0.01	0.48
	high > excess	3.31	<0.01	0.37
	low > high	2.46	0.02	0.24

### Discussion

**Figure 3:**
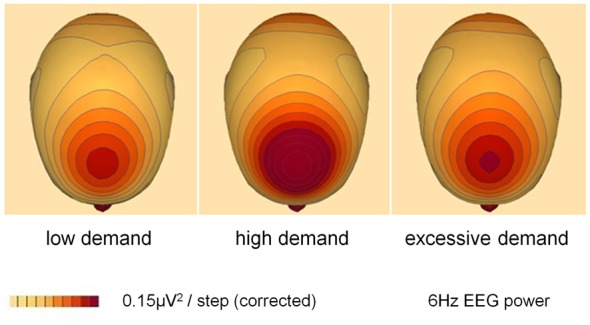
**Grand average topographic distribution of spectral power at the frequency of peak power for low, high and excessive demand (*N* = 20: incentive and no-incentive conditions are collapsed).** Peak frequency = 6 Hz (the frequency at which a clear peak in EEG power was evident within the 4–7 Hz range); this was identified by visual inspection of the grand average frequency-power spectral plot. Images were constructed using spherical spline interpolation.

**Figure 4:**
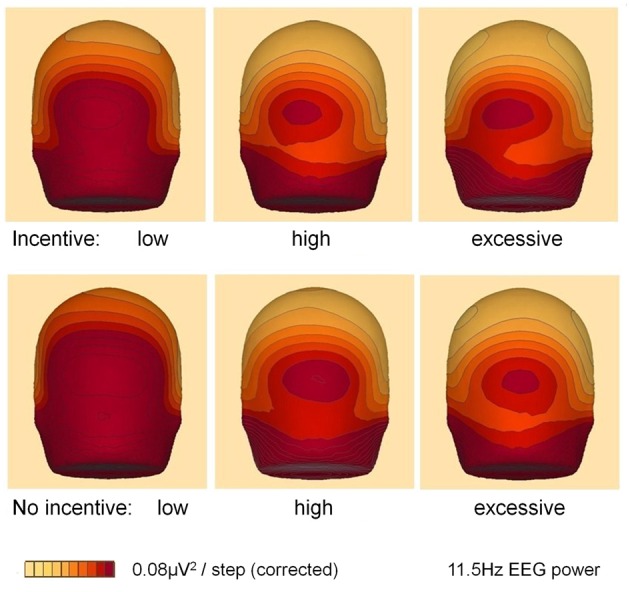
**Grand average spectral electroencephalography (EEG) power at 11.5 Hz (*N* = 20) for low, high and excessive cognitive demand on the Tetris game with and without a game based incentive (spherical spline interpolated; image displays rear of scalp)**.

This study was performed to assess the suitability of oscillatory EEG metrics for the real time monitoring of effort and cognitive demand during Tetris play. The results indicated frontal theta was robustly sensitive to objective game demand but that alpha activity only responded to demand at specific sites. For both frontal theta power and subjective motivation there were significant quadratic trends with maxima at high demand indicating that this level stimulated the highest subjective motivation and effort investment, as predicted by the MIM (Figures 1, 3). Upper alpha band (10.5–13 Hz) indicated a linear increase in cortical activation as the challenge of the game increased (Figure 4), which corresponded with the trend in subjective workload (Table 2). There was no main effect for either manipulation upon the lower alpha band (7.5–10 Hz) however, an interaction with site revealed sensitivity to demand over temporal and occipital areas of the scalp. The sensitivity of upper alpha activity to game demand was specific to frontal, central and parietal sites. In addition, upper alpha was the only frequency band to respond to the incentive coins (greater power reduction when game coins were present over the right hemisphere).

Augmentation of frontal theta has been widely reported in association with sustained attention, increased cognitive control and working memory (Gevins et al., 1998; Klimesch, 1999; Jensen and Tesche, 2002; Gevins and Smith, 2003; Sauseng et al., 2005; Cavanagh and Frank, 2014; Hsieh and Ranganath, 2014; Clayton et al., 2015). However, the decline of frontal theta power under conditions of excessive demand (Figure 3) has not previously been observed. The reproduction of this pattern in Tetris players provided an indication of the ecological validity of this metric and the ability of frontal theta to retain sensitivity to demand when generalized to spatial cognition in a gaming context. The capacity of frontal theta to act as a “generic” index of mental effort makes it an appropriate input to a closed-loop system since games typically use different elements of cognition at different stages of play. In addition, frontal theta demonstrated a degree of face validity owing to the similar pattern of modulation between EEG activity in this band and subjective motivation. The large effect sizes attest to the sensitivity of this measure and its capacity to discriminate between three or more categories of demand as well as detect the “tipping point” where effort is withdrawn due to overload (Figure 1).

Alpha power in the upper band, which is associated with task-specific cognitive processes (Klimesch, 1999), was suppressed as demand increased from low to high to excessive levels (Figure 4); a finding supported by a significant body of literature on cortical activation (e.g., Pfurtscheller, 1992; Gevins et al., 1998; Fournier et al., 1999; Klimesch, 1999). However, this main effect did not extend to lower band power (an index of cortical arousal and alertness), instead an interaction between demand and site showed that sensitivity of lower alpha band was limited to occipital and temporal areas. The lessening of power in the upper alpha band, and hence the level of cortical activation, was maximal during excessive demand despite a reduction of frontal theta power at this level. This suggests that upper alpha reflected the objective level of task demand upon spatial cognition (e.g., the processing of high numbers of fast moving stimuli in the form of falling Tetris blocks) whereas frontal theta represented the level of effort mobilization in the face of excessive demand (i.e., a withdrawal of effort). These findings suggested that a two-dimensional space could be created akin to the MIM (Figure 1) wherein demand is represented by upper alpha power and frontal theta power is used as an index of mental effort.

The sensitivity of the alpha band was found to vary across recording sites. Upper band effects occurred at frontal, central and parietal sites which provides some agreement with other studies linking these cortical areas with mental rotation—a key cognitive component of Tetris play (e.g., Inoue et al., 1998; Yoshino et al., 2000). Conversely, the effects of demand in the lower alpha band were restricted to temporal and occipital electrodes. In addition, the lower band revealed stronger activation in right hemisphere, which is traditionally associated with spatial tasks (Hellige, 1993), whereas the upper band indicated bilateral sensitivity to game demand. This regional variation indicates the importance of targeting the right cortical sites in order to maximize the sensitivity of the EEG metrics to the chosen psychological variables.

The results from the study identified two EEG measures as suitable inputs to a biocybernetic loop designed to control an adaptive game of Tetris. Frontal theta was selected to index mental effort due to its sensitivity to this variable, its reliability and its specificity (i.e., theta did not respond to the incentive+feedback manipulation), The sensor location Fz, which generally lies at the center of the scalp area associated with frontal theta augmentation was selected as the recording site. Power in the upper alpha band (10.5–13 Hz) was selected to index the level of task cognition; this variable was sensitive to the objective difficulty of the task and demonstrated a linear pattern over the three levels of demand in accordance with subjective workload ratings. There is also strong literature based support for the involvement of upper alpha band with task related cognition, including mental rotation (for a review see Klimesch, 1999). The right parietal site P4 was the chosen sensor input for the sampling of upper alpha oscillations. A parietal site was selected because in the first study parietal sites P3 and P4 detected sensitivity to game demand; central sites were also responsive but there were concerns that these would be subject to confounds from motor activity associated with game play. Although sensitivity was recorded at frontal sites this was smaller in magnitude than the parietal response to demand (Table 4). The choice of recording site was also constrained to the set of sites analyzed in study one i.e., frontal (F3, F4), temporal (T7, T8), central (C3, C4), parietal (P3, P4) and occipital (O1, O2) to preserve the validity of the psychophysiological inference regarding game-related cognition. Although there was no interaction of demand with hemisphere in the first study to guide this selection of site, the right hemisphere electrode P4 was selected on the basis of a robust association of right hemisphere with spatial cognition (Klimesch, 1999).

**Figure 5:**
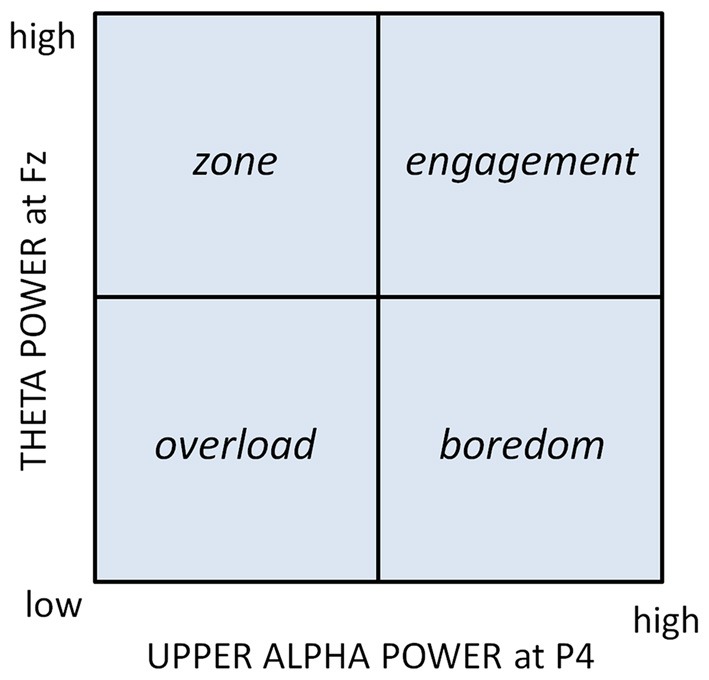
**Two dimensional representation of the user state using EEG measures (cortical activation is inversely proportional to alpha band power)**.

To summarize, the selection of the two EEG inputs to the biocybernetic loop made it possible to operationalize the adapted MIM (Figure 1) i.e., frontal theta was used to represent effort and parietal upper alpha to represent game demand (Figure 5). According to this conceptual model the desirable states of “zone” and “engagement” are associated with high effort while undesirable states are defined by low effort combined with high demand (overload) or combined with low demand (boredom).

## Development of the Real-Time Adaptive Gaming System

**Figure 6:**
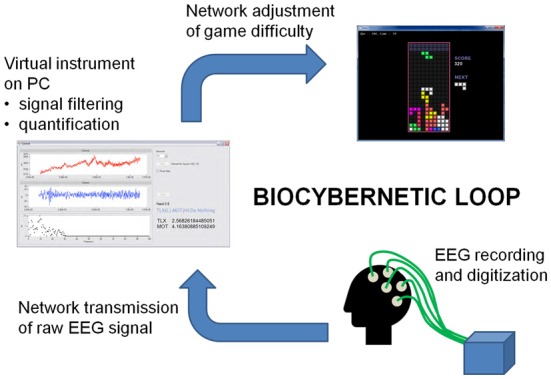
**Components of the biocybernetic loop**.

The working biocybernetic loop was created from a network that involved the connection of two PCs; one PC that ran the adaptive Tetris Software and a second PC that hosted a virtual instrument (VI) constructed with LabVIEW. Raw EEG data were transmitted to the VI to be filtered and averaged prior to transformation into estimates of motivation and workload by a state classification algorithm. These estimates were defined in terms of the four states of boredom, engagement, zone and overload (Figure 5). If the state fell within the undesirable categories of boredom or overload, a signal would be transmitted to the adaptive Tetris Software in order to adjust the level of game demand. The components of this loop are illustrated in Figure 6.

EEG data was recorded monopolarly from two Ag-AgCl pin-type active electrodes mounted in a BioSemi head cap at the locations Fz and P4 (sites determined by the 10–20 system). AC differential amplifiers amplified signals at source with continuous digitization at 16,384 Hz and online down sampling to 512 Hz. No filters were applied online to allow visual inspection of noise. The EEG signal was filtered using a Kasier Finite Impulse Response (FIR) of 2–30 Hz then subjected to a FFT in real time using a 2 s Hanning window. Theta activity between 4–8 Hz was obtained from the midfrontal electrode Fz and activity in the upper alpha band (10.5–13 Hz) was derived from right parietal site P4. The FFT calculated power spectra for each frequency band to generate total power values for each measure. These values were then converted to estimates of workload (upper alpha) and motivation (frontal theta).

For the operational model to trigger adaptations of game demand in real-time, it was necessary to select criteria for adaptation so that the four regions of the user state model could be defined (Figure 5). To maximize the effectiveness of adaptation, it was desirable to calibrate the criteria or trigger levels to individual players to counteract individual variability in the magnitude of EEG responses to game demand (Gevins et al., 1998).

The criteria for triggering adaptations of the Tetris interface were developed based upon patterns of theta and upper alpha oscillations that were observed relative to a baseline reading. Our participants were required to watch a relaxing video clip (Piferi et al., 2000) in order to establish baseline EEG levels of frontal theta and (parietal) upper alpha for each participant. Baselined derivatives of theta and alpha were captured in 5-s windows during subsequent game play. For example, if frontal theta activity increased or decreased from baseline by 100% in any 5-s window whilst parietal alpha increased or decreased by 100% then system adaptation may be triggered. In practice, frontal theta and parietal alpha were assessed every 5 s as the participant played the adaptive version of Tetris. If the system detected that frontal theta had decreased by 100% or more (from baseline) whilst parietal alpha had increased by 100 or more (from baseline), the player was assessed to be in a state of boredom (Figure 5). If the decrease of frontal theta was accompanied by a decrease of parietal alpha, the player was deemed to be in a state of overload.

A straightforward strategy for the adaptation of the game interface was used, i.e., reducing or increasing the drop speed of the falling Tetris blocks to manipulate game difficulty. Speed was increased if the player was deemed to be in a state of boredom and decreased if overload was detected (Figure 5). If neither of those states were detected by the system, the drop speed of the Tetris blocks was maintained. This assessment took place in 5-s epochs, hence the drop speed of the game increased or decreased over a period of play depending on the relative frequency of “boredom” or “overload” epochs that occurred within that period.

A series of pilot tests were conducted to determine an appropriate magnitude of the drop speed changes and whether or not to incorporate feedback of drop speed into the interface. The outcomes from these tests indicated that small adjustments without any overt feedback of drop speed were the most acceptable version of the Tetris interface from a user perspective. This design corresponded to a covert adaptive strategy where the adaptive process is expected to produce a gradual impact rather than an immediate impact on player state. This strategy was adopted in order to focus the attention of the players on the game as opposed to the ongoing activity of the biocybernetic loop.

## Study Two: Evaluation of the Biocybernetic Loop

### Introduction

A study was conducted to evaluate the adaptive Tetris game with respect to two questions: (1) does adaptation improve player experience compared to a manual adjustment of game demand; and (2) how does varying the reactivity of the biocybernetic loop (i.e., liberal vs. conservative trigger levels) impact upon player experience and the behavior of the closed-loop. The first question contrasts a covert, automated process of adjustment with a scenario where adjustments of game demand are both overt and manually instigated by the player. The second question pertains to the design of the trigger events for adaptation and how psychophysiological criteria can impact upon the process of system adaptation and the player experience.

### Method

#### Design

Three types of biocybernetic loop were compared: (a) a conservative system that produced an upward or downward adjustment of game demand (i.e., drop speed) when changes in frontal theta and parietal alpha substantially deviated from baseline (greater than 200%); (b) a liberal system that adjusted game demand in response to smaller deviations from baseline EEG activity (100%); and (c) a moderate system that responded to moderate changes in EEG (150%). It was anticipated that the conservative system would be the least reactive and would respond slowly and only to extreme examples of boredom and overload. By contrast, the liberal system was expected to make frequent adjustments and be the most responsive to instances of boredom/overload. For the fourth system, which operated under manual control, participants were required to speak aloud an instruction to increase (“higher”) or decrease (“lower”) the speed of the falling blocks. These adjustments were made in real-time by an experimenter sitting behind a screen in the laboratory. Ten participants played each of the four Tetris games (conservative closed-loop, liberal closed-loop, moderate closed-loop, manual) for 5 min. The order of presentation of each system was counterbalanced and participants were given a 5 min rest break between each game. Every game began on the slowest speed setting. If the blocks reached the top of the board and “game death” occurred, the game would restart with an empty board on the slowest speed setting. The procedure for the experiment and data collection protocol was approved by the LJMU University Research Ethics Committee and the experiment was conducted in accordance with the recommendations of this same committee.

#### Participants

Ten volunteers (6 females) participated in the evaluation session. A repeated measures design was used where each participant encountered each of the four versions of the system (conservative/liberal/moderate/manual). All participants were volunteers who gave their written informed consent prior to data collection in accordance with the Declaration of Helsinki.

#### Subjective Measures

Player experience was analyzed using subjective measures of mood and game immersion. The mood adjective checklist (UMACL; Matthews et al., 1990) assesses three components of mood: energetical arousal (EA: tired-alert), tense arousal (TA: relaxed-tense) and hedonic tone (HT: happy-sad). The UMACL was administered before and after each game to allow calculation of the change scores (post- minus pre-game) for each mood component. Participants also completed the Immersive Experience Questionnaire (IEQ) designed to capture the immersive quality of the gaming experience (Jennett et al., 2008); this scale was administered after each game.

#### Measures of System Behavior

Data were obtained in order to quantify the behavior of each version of the system. This enabled the three versions of the adaptive closed-loop to be contrasted with one another and an understanding to be acquired of how they differed from the manual control system. Three aspects of system behavior were measured for each system version:

- The mean frequency of increases and decreases in game demand
- The mean frequency of game deaths/resets (when blocks reached the top of the board and the game required resetting)
- The average demand level of each game (game difficulty could vary between 1 and 10 in accordance with the drop speed of falling blocks)

### Results

An ANOVA analysis was conducted on each of the three measures of system behavior (see Table 5 below for the descriptive statistics). Each measure (mean freq. of increases/decreases in demand, mean freq. of game deaths (resets), average game difficulty) was subjected to a one-way ANOVA to assess statistical significance. The number of adjustments to increase task demand was significantly higher for the conservative system compared to the other three systems; unsurprisingly, all three biocybernetic systems exhibited a higher rate of upward adjustment compared to the manual system (*F*_(3,7)_ = 79.40, *p* < 0.01). The analysis of downward adjustment (to decrease game demand) revealed that automated decreases of demand occurred more frequency during games played with the moderate and liberal versions of the biocybernetic loop (*F*_(3,7)_ = 18.4, *p* < 0.01). The analysis of reset frequency indicated that resets were most common in the conservative system, however, this increase failed to reach statistical significance. The analysis of mean demand level indicated that difficulty was significantly lower for the liberal system compared to all other systems (*F*_(3,7)_ = 12.3, *p* < 0.01).

**Table 5:** **Mean values for measures of system adaptation across the four systems (*N* = 10)**.

System	Increase demand	Decrease demand	Mean reset	Mean difficulty level
Conservative	63.6	43.2	1.3	3.8
Moderate	41.7	56.6	0.6	2.4
Liberal	28.6	62.4	0.4	1.9
Manual	9.4	1.7	0.5	3.3

*Highest difficulty level = 10; lowest difficulty = 1*.

The impact of system adaptation on the user experience was assessed using two types of subjective questionnaire; the IEQ and the UMACL mood adjective checklist. The UMACL was administered before and after each game session in order for us to calculate a change score that quantified the changes in the three components of mood: EA (alert-tired), TA (tense-relax) and HT (happy-sad). All three components were subjected to a one-way ANOVA; mean values are displayed in Table 6.

**Table 6:** **Mean values for subjective data: EA, energetic arousal (change score); TA, tense arousal (change score); HT, hedonic tone (change score); IEQ, immersion (*N* = 10)**.

System	EA	TA	HT	IEQ
Conservative	4.3	3.1	0.0	64.7
Moderate	2.0	2.4	−1.9	65.5
Liberal	0.2	1.1	−2.0	66.1
Manual	1.1	0.7	−1.6	73.9

The mean values for the changes in mood indicated some consistent trends, namely that participants found the game to be alerting and conducive to tension and negative affect. An ANOVA analysis of all three mood components revealed a significant effect for EA (energetic arousal) only (*F*_(3,7)_ = 5.48, i.e., *p* < 0.05), i.e., participants found the experience of playing the conservative version of the biocybernetic game to be more alerting compared to the liberal version (*p* < 0.05). The analysis of responses to the immersion questionnaire was insignificant, but a trend was observed that participants found the manual version of the game to be the most immersive.

### Discussion

This evaluation study demonstrated how the reactivity of the biocybernetic loop affected the performance of the system and the experience of players.

The analysis of system behavior revealed that the conservative system provided the greatest level of challenge, i.e., it produced the highest average level of demand and made the largest number of adjustments to increase game demand. This skew towards increased adjustment of demand was mirrored by the liberal system, which tended to adjust difficulty in the opposite direction, such that the liberal version produced the lowest number of game deaths and lowest average level of demand. The moderate system produced a pattern of upward and downward adjustments that represented a midpoint between that of the conservative and liberal systems. As anticipated, the number of adjustments made manually by participants was lower than the numbers produced by the biocybernetic loop as they tended to simply increase the level of difficulty to their preferred level early in the game without making any subsequent adjustments. The mean level of difficulty during play on the manual system (included as benchmark to compare with the adaptive protocols) provided an indication of the optimal level of demand for the group (3.3). By contrast the conservative system generally pushed the players to a higher level of demand (3.8), resulting in the greatest number of game deaths; the moderate and liberal systems tended to set difficulty at a lower level than the manual system on average. Therefore, the three adaptive systems and their respective triggers tended to either over- or undershoot the mean level of demand that was preferred by our participants.

It was noteworthy that the conservative system produced a large number of upward adjustments in game demand (63.6) suggesting that this system was detecting boredom via the EEG (i.e., a 200% decrease in theta and increase in alpha relative to baseline; Figure 5). Boredom may have resulted from games starting on the slowest drop speed setting and the return to the slowest speed when the game was reset (Table 5). By contrast, the liberal system produced more downwards than upwards adjustments even though games started at the easiest level, meaning on some occasions where the trigger criteria for a downward adjustment was fulfilled the interface was unable to slow the speed because the player was already at the lowest level. The liberal system was also detecting more player overload than the other two adaptive systems (more downward adjustments), i.e., the trigger criteria of a 100% decrease in theta and alpha power relative to baseline were fulfilled the most frequently (Figure 5). This is surprising in view of the low levels of demand delivered by the liberal system. One explanation may be that the EEG indicators of overload used were incorrect and that simultaneous decreases in alpha and theta power of around 100% indicate low effort (reduced frontal theta) combined with low levels of sensory processing (reduced parietal alpha) instead of overload (Klimesch, 1999). It may be that deviations of 200% or more are required to indicate overload where excessive demand leads to a high level of alpha power suppression, as occurred when demand was excessive in the first study (Figure 4). This underlines the importance of not only selecting the best combination of input measures for the biocybernetic loop, but also of defining accurate trigger criteria in terms of the relative magnitudes of the input variables.

It was expected that player experience would be affected by the different outcomes in system behavior between the four versions of the system. However, there were few statistically significant effects on mood and immersion. Alertness was enhanced under the conservative system relative to the liberal system but there were no other significant effects. Of the four systems analyzed the conservative version of the loop produced the most desirable overall impact on player mood, i.e., it evinced the greatest increase in arousal and least negative affect which may be because participants were too challenged to dwell upon their emotional state. This may be because the conservative system was the most successful at detecting boredom and alleviating it with increases in demand. Conversely ratings of immersion in the game were greatest for the manual system, which may reflect the impact of taking momentary breaks from the game to voluntarily control difficulty with a verbal instruction. Even very short breaks from a task are known to increase vigilance performance (Ariga and Lleras, 2011) and opportunities for control can increase the intrinsic motivation for a task (Fisher, 1978). Alternatively, it may be argued the level of demand during the manual control condition was optimal for enhancing immersion. The observation that play on the game increased negative affect under all but the conservative system was unanticipated and may reflect the impact of the lower levels of challenge experienced by participants.

Based upon the results, it would appear that the criteria used to define the three versions of the biocybernetic loop were too similar to evince much difference in player experience. It may be speculated that if players were provided with more time to experience play upon each version of the system they may have been better able to differentiate their respective experiences.

The biocybernetic loop employed a straightforward linear process of calibration to the individual instead of machine learning algorithms. The rationale for this approach was that our psychophysiological measures, EEG theta and upper alpha frequency power, had been validated prior to construction of the loop—and we wished to preserve the transparency of both measures and criteria when testing the working loop. However, there may have been scope to use machine learning during calibration such that more precise linear models may have been generated especially for each participant.

The results highlighted a number of questions surrounding the evaluation of working biocybernetic systems, particularly with respect to the benchmarking of system performance. In this study, a manual system was selected as the benchmark for comparative purposes on the assumption that participants would tailor gameplay to their personal preference. However, this comparison was asymmetrical because the locus of system control for a manual system resided with the user while control was automated within the biocybernetic loop. This is a significant factor when comparing player experience across automated and manual systems since the opportunity for control over a task (as provided by the manual system) is known to affect the level of engagement with that task (Fisher, 1978; Wright and Kirby, 2001). Comparisons with other autonomous systems may therefore be more informative. For example, benchmarking against a system that adapts game demand in a random fashion without an objective rationale, or by using a “yoked” system where the game responds to the physiology of another individual (Bailey et al., 2006). Either of these options may have provided a more parsimonious comparison with the three versions of the working biocybernetic loop.

## General Discussion

This article has described the process of creating a working biocybernetic loop whereby hypotheses derived from experimental work on EEG were first validated in a gaming context in order to select the input measures for the loop. Predictions regarding the modulation of EEG frontal theta and alpha power by variations in the level of cognitive demand and effort were validated during Tetris play; subsequently an adaptive game of Tetris was built that used a biocybernetic loop with the EEG measures tested during the validation stage. Our development process for this prototype exemplifies the principle of designing interactive technologies based upon a theory-driven process of psychophysiological inference (Fairclough, 2009).

The evaluation of autonomous, closed-loop control systems raises important issues for the development of biocybernetic adaptation. The relationship between criteria or categories of psychophysiological activity and the triggering of adaptive responses at the interface requires careful design. The derivation of valid input measures and effective categorization of psychophysiological data in real-time is one stage of this process. Once a method of categorizing the states of the user has been defined (Figure 5), these classes must be mapped onto appropriate responses at the interface. This mapping reflects more than a simple linkage between state *x* and response *y*; decisions must be made regarding the frequency and likelihood of those responses as well as the temporal characteristics and relative magnitude of the adaptations. As was demonstrated in the evaluation study, once a working biocybernetic loop has been constructed, responses may be adjusted to optimize the user experience, a process that inevitably involves exploring the interaction between the user and the adaptive response. The behavior of the biocybernetic loop and the interaction between user psychophysiology and adaptive control is an object of study in itself.

Together these two studies provide a potential blueprint for the development and evaluation of a biocybernetic loop. However, further research is required to incorporate psychophysiological theory into the design of physiological computing systems and to develop an effective methodology for system evaluation.

## Author Contributions

The design and experimental protocol used for Study 1 was developed by KCE and SHF. The Tetris game used in Study 1 was built by KG. Data collection and analysis for Study 1 was performed by KCE. The design and experimental protocol for Study 2 was developed by KG and SHF. Build of the biocybernetic loop and adaptive game of Tetris for Study 2 was performed by KG as was the data collection and analysis for this study. The manuscript was written and edited by KCE (primary author), SHF and KG.

## Funding

This research was funded by a grant from the REFLECT Project; the European Union’s Future and Emerging Technologies Scheme, 7th Framework Programme (FP7). Grant number 215893. Project website: http://reflect.pst.ifi.lmu.de/.

## Conflict of Interest Statement

The authors declare that the research was conducted in the absence of any commercial or financial relationships that could be construed as a potential conflict of interest.
